# Supplementary material for: Reproducible and accessible analysis of transposon insertion sequencing in Galaxy for qualitative essentiality analyses
Source: BMC Microbiol. 2021 Jun 5;21:168. doi: 10.1186/s12866-021-02184-4 (PMC8178898; doi:10.1186/s12866-021-02184-4)
Supplement: Supplementary file 1 — Additional file 1 Supplementary file. [file 12866_2021_2184_MOESM1_ESM.pdf]

Additional data

Tables

Threshold	$log_2(4)$	$log_2(12)$
Essential	369	364
Non-Essential	3822	2111
Undetermined	112	1828

Additional Table 1: Gene essentiality prediction of regression for both thresholds, Tn5 with E.coli.

Threshold	$log_2(4)$	$log_2(12)$
Essential	480	414
Non-Essential	2324	2262
Undetermined	37	165

Additional Table 2: Gene essentiality prediction of regression for both thresholds, Himar1 and S.aureus.

Figures

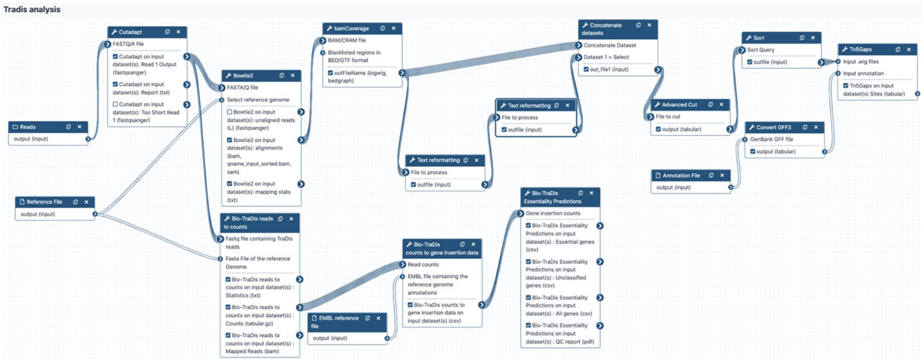

Figure 1: **Tradis - Galaxy Workflow.**

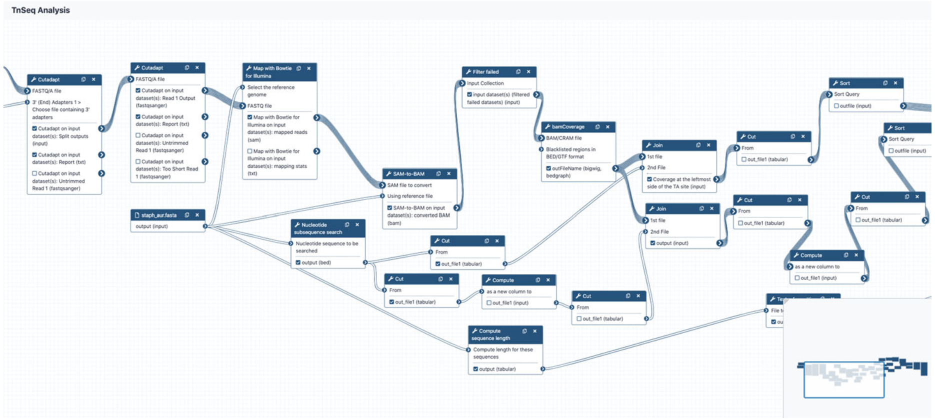

Figure 2: **TnSeq - Galaxy Workflow.**

Information

- Workflow for Tradis Analyses : https://usegalaxy.org/u/delphinel/w/tradis-analysis
- Workflow for TnSeq Analyses : https://usegalaxy.org/u/delphinel/w/tnseq-analysis

- GitHub repository of the paper : https://github.com/galaxyproject/TIS_methods_review
- Training material for TIS https://training.galaxyproject.org/training-material/topics/genome-ann[illegible]
- Detail of Regression on TnSeq Data : https://github.com/galaxyproject/TIS_methods_review/blob/mast[illegible]
- Detail of Regression on TnSeq Data : https://github.com/galaxyproject/TIS_methods_review/blob/mast[illegible]
